# Oxidative Status and Antioxidative Response to *Fusarium* Attack and Different Nitrogen Levels in Winter Wheat Varieties

**DOI:** 10.3390/plants10040611

**Published:** 2021-03-24

**Authors:** Magdalena Matić, Rosemary Vuković, Karolina Vrandečić, Ivna Štolfa Čamagajevac, Jasenka Ćosić, Ana Vuković, Kristina Sabljić, Nikolina Sabo, Krešimir Dvojković, Dario Novoselović

**Affiliations:** 1 Faculty of Agrobiotechnical Sciences, Josip Juraj Strossmayer University of Osijek, Vladimira Preloga 1, 31000 Osijek, Croatia; maticm@fazos.hr (M.M.); jcosic@fazos.hr (J.Ć.); 2 Department of Biology, Josip Juraj Strossmayer University of Osijek, Cara Hadrijana 8/A, 31000 Osijek, Croatia; ivna@biologija.unios.hr (I.Š.Č.); ana.vukovic@biologija.unios.hr (A.V.); kristina.sabljic@biologija.unios.hr (K.S.); nikolina.sabo@biologija.unios.hr (N.S.); 3 Centre of Excellence for Biodiversity and Molecular Plant Breeding (CoE CroP-BioDiv), 10000 Zagreb, Croatia; darion@poljinos.hr; 4 Department for Cereal Breeding and Genetics, Agricultural Institute Osijek, Južno Predgrađe 17, 31000 Osijek, Croatia; kresimir.dvojkovic@poljinos.hr

**Keywords:** *Triticum aestivum*, *Fusarium*, nitrogen, oxidative stress, antioxidant system

## Abstract

Abiotic and biotic stresses, such as mineral nutrition deficiency (especially nitrogen) and *Fusarium* attack, pose a global threat with devastating impact on wheat yield and quality losses worldwide. This preliminary study aimed to determine the effect of *Fusarium* inoculation and two different nitrogen levels on oxidative status and antioxidative response in nine wheat varieties. Level of lipid peroxidation, activities of antioxidant enzymes (catalase, ascorbate peroxidase, glutathione reductase), phenolics, and chloroplast pigments content were measured. In general, wheat variety, nitrogen, and *Fusarium* treatment had an impact on all tested parameters. The most significant effect had a low nitrogen level itself, which mostly decreased activities of all antioxidant enzymes and reduced the chloroplast pigment content. At low nitrogen level, *Fusarium* treatment increased activities of some antioxidative enzymes, while in a condition of high nitrogen levels, antioxidative enzyme activities were mostly decreased due to *Fusarium* treatment. The obtained results provided a better understanding on wheat defense mechanisms against *F. culmorum,* under different nitrogen treatments and can serve as an additional tool in assessing wheat tolerance to various environmental stress conditions.

## 1. Introduction

Wheat (*Triticum aestivum* L.) is one of the most important cereal crops worldwide. In field conditions, wheat is confronted with both abiotic and biotic stresses that have a great impact on its growth and productivity [1]. Nitrogen (N) is one of the major nutritional elements in wheat production, and it is necessary to achieve high yields and grain quality [2]. Wheat quickly perceives and responds to nitrogen deficiency via a large number of physiological and metabolic events, such as the changes in fatty acid composition, reduction in chlorophyll content, and occurrence of oxidative stress [3]. During cultivation, wheat is often exposed to Fusarium head blight (FHB), an economically devastating disease that may exert an adverse impact on the wheat yield and quality [4]. Global yield losses due to individual pathogen and pests are estimated at 21.5% for wheat, whereas yield losses due to FHB ranked second after leaf rust [5]. FHB is caused by species of fungi in the genus *Fusarium*, of which *Fusarium graminearum* Schwabe (*Gibberella zeae* Schwein. Petch.) and *Fusarium culmorum* (Wm. G. Sm.) Sacc. are the most common and most virulent in Croatia [6].

It is now well established that all abiotic and biotic stresses induce or involved oxidative stress to some degree, and the ability of plants to control oxidant levels is highly correlated with stress tolerance [7]. Oxidative stress is a complex chemical and physiological phenomenon that arises due to excessive production and accumulation of reactive oxygen species (ROS) and reactive nitrogen species (RNS) [8,9]. The balance between production and elimination of ROS and RNS is critical to maintaining cellular redox homeostasis. Excessive production of ROS and RNS can induce a nitro-oxidative stress in plant cells, which causes lipid peroxidation, damages proteins and nucleic acids, inhibits antioxidant enzyme activities and activates the programmed cell death pathway [8,10]. In order to overcome the high levels of ROS and RNS, plants developed an efficient antioxidant defense system constitutes both enzymatic and nonenzymatic components. Enzymatic components that are involved in the detoxification of reactive species include superoxide dismutase (SOD), catalase (CAT), ascorbate peroxidase (APX), and glutathione reductase (GR), while the non-enzymatic components include small molecules such phenolics (PHE), flavonoids, pigments, ascorbate, and glutathione [8,11].

Plant tolerance to different stress factors can be achieved by plant breeding or cultural practices that reduce levels of stress, which is in turn accomplished by the understanding of the plant’s response to its stressors and how they affect individual plants and plant processes [12]. This study aimed to determine the effect of two different nitrogen levels and *Fusarium* inoculation on the biomarkers of oxidative and antioxidative status (lipid peroxidation, PHE, and activities of CAT, APX, and GR) measured in the spikes of nine wheat varieties. Although numerous studies have analyzed wheat’s physiological response to *Fusarium* stress itself [13,14,15], this preliminary study will give insight into wheat’s physiological response to combined nitrogen and *Fusarium* stress conditions.

## 2. Results

Three-way ANOVA showed significant differences in visual FHB scoring for all the main factors (Table 1). The visual scoring for FHB was affected mainly by *Fusarium* treatment and wheat variety (*p* ≤ 0.001) and to a lesser extent by nitrogen treatment (*p* ≤ 0.05). Non-inoculated wheat varieties did not show any FHB symptoms; thus statistical differences between inoculated and non-inoculated plants are not shown. Differences in symptoms of inoculated wheat varieties at low and high nitrogen levels for each variety separately are shown (Table 2). In Ficko, Galloper, and Felix varieties high nitrogen level caused an increase in visual symptoms. Contrarily, in variety U-1 high nitrogen level caused a decrease in visual symptoms.

Three-way ANOVA revealed a significant variety, nitrogen and *Fusarium* treatment effects for tested biochemical parameters (Table 1). Wheat variety significantly affected all tested biochemical parameters (*p* ≤ 0.001), while nitrogen treatment significantly affected thiobarbituric acid reactive substances (TBARS) content, CAT and APX activity. *Fusarium* treatment mostly affected PHE content (*p* ≤ 0.001), and to a lesser extent, CAT and GR activity (*p* ≤ 0.05). Variety by nitrogen treatment interaction was significant for TBARS, PHE accumulation, and APX activity. Three-factor interaction between the variety, nitrogen, and *Fusarium* treatment was significant only for CAT and APX activity.

**Figure 1:**
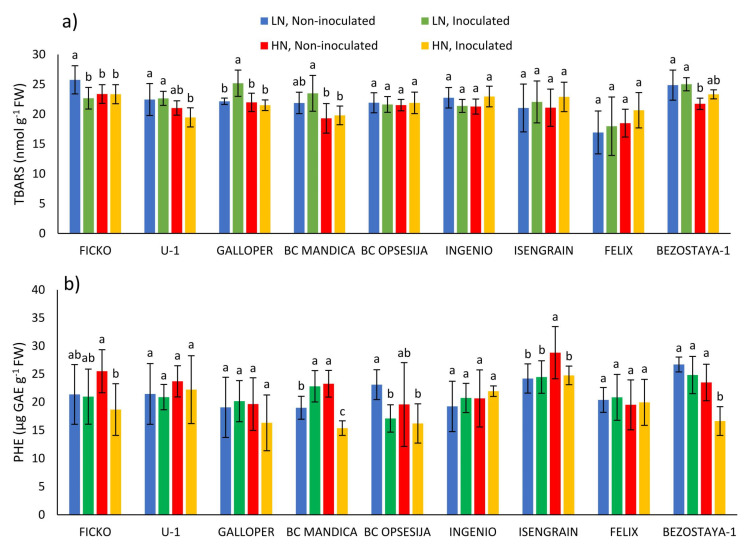
TBARS content (**a**) and soluble phenolic (PHE) content (**b**) in spikes of nine wheat varieties under different nitrogen (LN—low nitrogen; HN—high nitrogen) and *Fusarium* (non-inoculated and inoculated) treatments. Values are means of six replicates ± standard deviation (SD). Different letters above the bars indicate significant differences according to Fisher’s LSD test (*p* ≤ 0.05) among different treatments in each variety separately.

TBARS content was significantly influenced by the wheat variety and nitrogen treatment (Table 1). In the Ficko variety at low nitrogen level, *Fusarium* treatment caused a decrease in TBARS content, while in the Galloper variety, the content of TBARS was increased (Figure 1a). In conditions of high nitrogen level, *Fusarium* treatment did not cause any significant changes in TBARS content. In most non-inoculated varieties, low nitrogen level tended to increase TBARS content, although a significant increase was only found in the Ficko and Bezostaya-1 varieties.

The soluble phenolic content was most significantly affected by *Fusarium* treatment and wheat variety (Table 1). *Fusarium* treatment caused an increase in soluble phenolic content in BC Mandica variety at low nitrogen level, while in the BC Opsesija variety this content was decreased (Figure 1b). In conditions of high nitrogen level, *Fusarium* treatment caused a decrease in phenolic content in the Ficko, BC Mandica, Isengrain and, Bezostaya-1 varieties. In most non-inoculated varieties, low nitrogen level decreased phenolic content, although a significant decrease was only found in the BC Mandica and Isengrain varieties.

**Figure 2:**
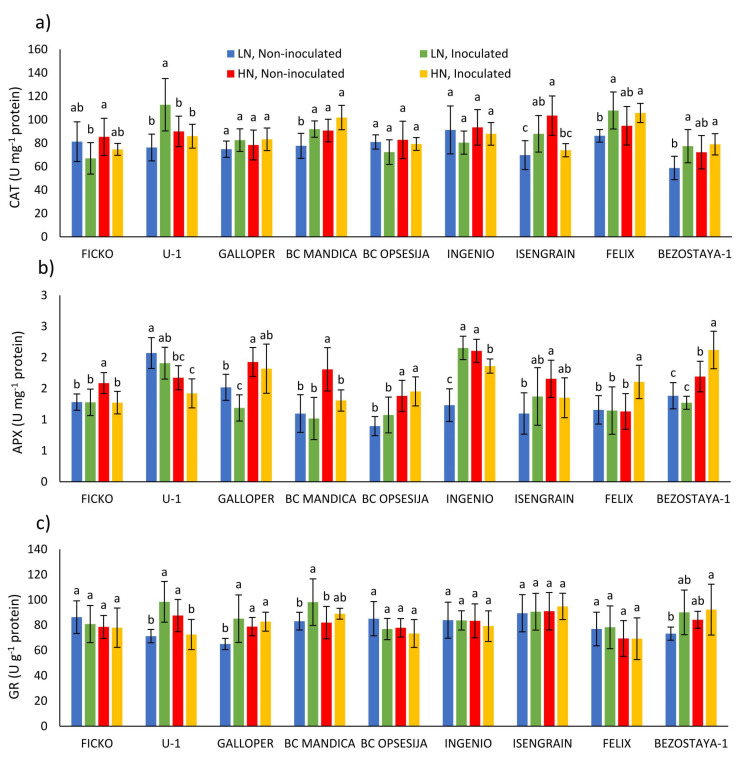
Antioxidant enzyme activity: catalase (CAT; (**a**)), ascorbate peroxidase (APX; (**b**)), and glutathione reductase (GR; (**c**)) in spikes of nine wheat varieties under different nitrogen (LN—low nitrogen; HN—high nitrogen) and *Fusarium* (non-inoculated and inoculated) treatments. Values are means of six replicates ± standard deviation (SD). Different letters above the bars indicate significant differences according to Fisher’s LSD test (*p* ≤ 0.05) among different treatments in each variety separately.

The CAT activity was significantly influenced by all three main factors, wheat variety (*p* ≤ 0.001), nitrogen (*p* ≤ 0.01), and *Fusarium* treatment (*p* ≤ 0.05; Table 1). On average, a low nitrogen level caused a decrease of 5.46% in CAT activity compared to a high nitrogen level. Observing the changes in CAT activity in each variety separately, in the U-1, BC Mandica, Isengrain, Felix, and Bezostaya-1 varieties, at low nitrogen level, *Fusarium* treatment caused an increase in CAT activity (Figure 2a). In conditions of high nitrogen level, *Fusarium* treatment caused a decrease in CAT activity in the Isengrain variety. In most non-inoculated varieties, low nitrogen level decreased CAT activity, although a significant decrease was found in the BC Mandica and Isengrain varieties.

The APX activity was most significantly influenced by nitrogen treatment and wheat variety (Table 1). On average, a low nitrogen level caused a decrease of 17.26% in APX activity compared to a high nitrogen level. Observing the changes in APX activity in each variety separately, in the Galloper variety at low nitrogen level, *Fusarium* treatment caused a decrease in APX activity, while in the Ingenio APX activity was increased (Figure 2b). In conditions of high nitrogen level, *Fusarium* treatment caused a decrease in APX activity in Ficko, BC Mandica, and Ingenio varieties, while in Felix and Bezostaya-1 varieties, APX activity was increased. In most non-inoculated varieties (Ficko, Galloper, BC Mandica, BC Opsesija, Ingenio, Isengrain, and Bezostaya-1), low nitrogen level significantly decreased APX activity, compared to high nitrogen level.

The GR activity was most significantly influenced by wheat variety (*p* ≤ 0.001), and to a lesser extent, *Fusarium* treatment (*p* ≤ 0.05; Table 1). In U-1, Galloper and BC Mandica varieties, at low nitrogen, *Fusarium* treatment caused an increase in GR activity, while, at high nitrogen conditions, GR activity was decreased only in U-1 variety (Figure 2c). Compared to high nitrogen level, low nitrogen level significantly decreased GR activity in non-inoculated U-1 and Galloper varieties.

Three-way ANOVA revealed a wide variety, nitrogen and *Fusarium* treatment effects for tested chloroplast pigments (Table 3). Wheat variety and nitrogen treatment significantly affected all tested chloroplast pigments in wheat spikes. *Fusarium* treatment significantly affected almost all tested chloroplast pigments, except the Chl a/Chl b ratio.

The strongest effect on chlorophyll a (Chl a), chlorophyll b (Chl b), total chlorophyll (Chl a+b), carotenoids (Car) content, and chlorophyll a/b ratio in wheat spikes was found in the nitrogen treatment (Table 3). On average, a low nitrogen level decreased Chl a, Chl b, Chl a+b, and Car content in wheat spikes, while chlorophyll a/b ratio was increased, compared to high nitrogen level. Wheat variety also had a strong effect on chloroplast pigment content in wheat spikes. On average for all varieties, Ingenio and Isengrain varieties showed the highest Chl a, Chl b, Chl a+b, and Car content in wheat spikes, while the lowest content of chloroplast pigments was in Bezostaya-1 variety (data not shown).

**Figure 3:**
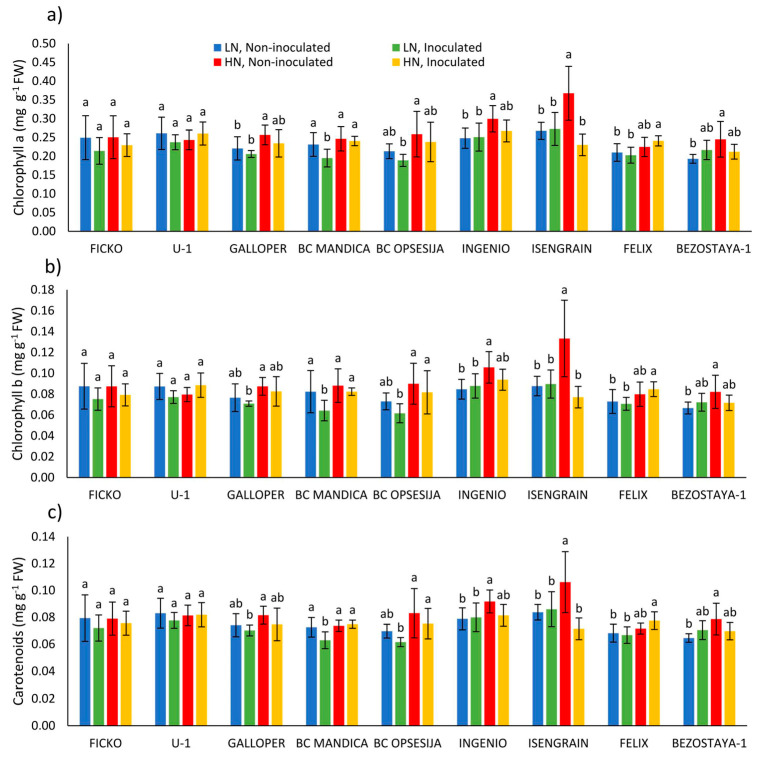
Chlorophyll a (**a**), chlorophyll b (**b**), and carotenoids (**c**) content in spikes of nine wheat varieties under different nitrogen (LN—low nitrogen; HN—high nitrogen) and *Fusarium* (non-inoculated and inoculated) treatments. Values are means of six replicates ± standard deviation (SD). Different letters above the bars indicate significant differences according to Fisher’s LSD test (*p* ≤ 0.05) among different treatment in each variety separately.

Observing the changes in chloroplast pigment content in each variety separately, *Fusarium* treatment, at both nitrogen levels, caused a decrease in Chl a, Chl b, and Car content in BC Mandica and Isengrain varieties (Figure 3a–c). In conditions of low nitrogen level, most non-inoculated varieties showed decrease in the content of chloroplast pigments compared to high nitrogen level. However, a significant decrease was found in Ingenio, Isengrain, and Bezostaya-1 varieties for Chl a, Chl b, and Car content, and for Chl a in the Galloper variety.

## 3. Discussion

Both inadequate nitrogen fertilization and pathogen attacks can cause abiotic and biotic stress conditions in wheat production. To date, a scarce amount of studies are available on the influence of both FHB and different nitrogen fertilization on the oxidative and antioxidant response of wheat. In the present study, we examined the effect of nitrogen and *Fusarium* treatment on the occurrence of FHB and biochemical changes in spikes of nine wheat varieties in field conditions. In general, wheat variety, nitrogen, and *Fusarium* treatment had an impact on all tested parameters of oxidative stress and antioxidative response.

Considering different experimental conditions, various reports on the effect of nitrogen fertilization on FHB are available in the literature. According to some studies, high nitrogen levels increase disease incidence and intensity [16,17,18], whereas others reported restriction impact of nitrogen on *Fusarium* infection [19,20,21]. In the present study, in, Ficko, Galloper, and Felix varieties high nitrogen levels caused an increase in visual symptoms, compared to low nitrogen levels. Contrarily, in U-1 variety high nitrogen level caused a decrease in visual symptoms, suggesting a variety-specific response. However, to determine a more accurate effect of different nitrogen levels on FHB incidence and intensity, it is necessary to include testing at different nitrogen levels and more extensive mycological, and mycotoxin analyses.

As an indicator of oxidative stress, lipid peroxidation can be a useful tool to evaluate cultivars’ susceptibility to FHB [14]. However, in our study, we did not find any significant changes in the levels of lipid peroxidation caused by nitrogen or *Fusarium* treatment. Under the nitrogen deficiency and *Fusarium* treatment, increased lipid peroxidation was recorded only in Galloper variety, which could be explained by varietal susceptibility to *Fusarium*. On the other hand, in conditions of high nitrogen level, *Fusarium* treatment did not cause any significant changes in lipid peroxidation level. This could be explained by different plant responses to individual stress compared to a combination of stresses [22,23,24].

In conditions of low nitrogen level, *Fusarium* treatment considerably increased activities of some measured antioxidative enzymes (CAT, APX or GR) in most varieties. The exception was the Galloper variety, the only variety with increased lipid peroxidation level, where APX activity was decreased, indicating the importance of this enzyme in the defense response. Induction of enzymatic defense mechanisms is connected with FHB-resistance, wherein more tolerant varieties activate the antioxidative enzymes faster and earlier in the infection process [25,26]. On the other hand, in conditions of high nitrogen levels, antioxidative enzyme activities were decreased in most infected wheat varieties. Although there were no changes in TBARS content, decreased activities of antioxidative enzymes could increase the incidence and intensity of FHB in conditions of high nitrogen levels. Different Fusarium impacts on antioxidative response in conditions of high and low nitrogen level could be due to different ROS formation and scavenging in conditions of single stress (*Fusarium* treatment) and combination of stresses (low nitrogen level and *Fusarium* treatment).

Polyphenols content, such as phenolic acids and flavonoids, increased under abiotic or biotic stress conditions, helping the plant to cope with environmental constraints [27,28]. In the present study, *Fusarium* treatment of wheat at low nitrogen level increased the phenolic content in BC Mandica variety, while in BC Opsesija variety phenolic content decreased in response to *Fusarium* treatment. Increased content of phenolics in BC Mandica variety could be a part of its defense response that could contribute against pathogen attack and spread. In conditions of high nitrogen level, *Fusarium* treatment of wheat decreased the phenolic content in Ficko, BC Mandica, Isengrain, and Bezostaya-1 varieties. When exposed to pathogen infection, plants often suffer significant chloroplast pigment loss [29]. In the present study, *Fusarium* treatment caused a decrease in all chloroplast pigments in BC Mandica and Isengrain varieties, in conditions of low and high nitrogen levels, respectively.

In this study, the most noticeable impact on the measured biochemical parameters in wheat spikes had a low nitrogen level itself. Various abiotic stress conditions lead to the overproduction of ROS and imbalanced ROS detoxification, which lead to lipid peroxidation [8,30]. The present study provided similar results, in which low nitrogen level as an abiotic stress factor caused an increase in lipid peroxidation in most varieties. Although a significant increase in lipid peroxidation was found only in non-inoculated Ficko and Bezostaya-1 varieties, which also showed reduced APX activities, indicating the importance of this enzyme in scavenging ROS. Moreover, in most varieties, low nitrogen level caused a decrease in antioxidant enzyme activities. Decreased activities of CAT, APX, and GR have also been demonstrated in other N-deficient plants [31,32]. The reduction in antioxidant enzymes activity could be connected with lower amino acid and protein synthesis in nitrogen deficiency conditions, compared to high nitrogen level conditions. In the present study, low nitrogen level caused a decrease in the soluble phenolic content in two varieties (BC Mandica and Isengrain). This finding is in contrast to other studies, in which the accumulation of phenolic components in plant tissues was enhanced under limited nitrogen conditions, due to increased C:N ratio within plants [33,34,35]. Although results were not shown in this study, phenolic contents were increased in flag leaves of most varieties, suggesting different tissue responses to nitrogen deficiency. Furthermore, low nitrogen level caused the reduction in chlorophyll a, chlorophyll b, total chlorophyll, and carotenoids content in wheat spikes in non-inoculated plants. Our findings are in accordance with previous studies in which decreased content of photosynthetic pigments under nitrogen-deficient conditions were found [3,36]. Low nitrogen level induces inhibition of photosynthesis and reduces photosynthetic capacity, which consequently inhibits plant growth and development [37,38].

The obtained results provided a better understanding on the biochemical aspects as a part of wheat defense mechanisms against necrotrophic fungi *F. culmorum,* under different nitrogen treatments. To our knowledge, this is the first report on the oxidative/antioxidative response of wheat to combined stress caused by *Fusarium* attack and different nitrogen levels. This research can serve as an additional tool in assessing wheat tolerance to various environmental stress conditions.

## 4. Materials and Methods

### 4.1. Field Trial

Field trial with nine winter wheat varieties of different origin (Table 4) was planted in 2017/18 season at location Osijek. The experiment was set-up in a split-split-plot-factorial design in three replicates with nitrogen fertilization levels as main plots, nine wheat cultivars as sub-plots, and *Fusarium* infection was applied at sub-sub-plot level.

The soil type was eutric cambisol and the experimental plot size was 7.56 m^2^. Basic fertilization of 74 kg N ha^−1^, 80 kg P_2_O_5_ ha^−1^ and 120 kg K_2_O ha^−1^ was applied by adding 100 kg ha^−1^ of urea (46% N) and 400 kg ha^−1^ NPK (7:20:30). The nitrogen treatment comprised of two nitrogen fertilization levels, 0 kg N ha^−1^ (LN − low nitrogen) and 100 kg N ha^−1^ (HN—high nitrogen), applied as top-dressings of 50 kg N ha^−1^ at tillering (Zadok’s scale 23–25) and stem extension (Zadok’s scale 33–35) growth stages (Table 5). All other cultural practices including the application of herbicides, insecticides, and fungicides to control major weeds, insects and foliar diseases were typical for commercial wheat production in Croatia.

### 4.2. Inoculum Production, Inoculation Procedure, and Disease Evaluation

Isolate *F. culmorum* obtained from the fungal culture collection of Faculty of Agrobiotechnical Sciences Osijek (Croatia) was used for inoculum production, according to the method of Snijders and Eeuwijk [39]. A mixture of wheat and oat grains (3:1, v/v) was soaked in water overnight. The following day, excess water was decanted and seeds were autoclaved and inoculated with *F. culmorum* isolate. Inoculated grains were incubated for three weeks at 25 °C, protected from sunlight. Macroconidia were washed off the colonized grains, and conidial suspension was diluted to a final concentration of 1 × 10^6^ conidia mL^−1^. Only the first m^2^ of each subplot (variety) was inoculated, while the rest of the subplot was left to natural infection. Spray inoculations were carried out using a motor-driven backpack-sprayer in the late afternoon. Inoculations were performed individually on each subplot when 50% of the plants had reached anthesis (Zadok’s scale 65) and repeated two days later. To maintain moisture for optimal infection conditions, plants were sprayed with water several times during the day. Visual scoring of the overall percentage of inoculated spikes showing FHB symptoms was performed 18 days after inoculation. The percentage of diseased spikes in each plot was determined on a linear scale (0–100%) [40].

### 4.3. Sample Preparation and Measurements

Wheat spikes for measuring biomarkers of oxidative stress and antioxidant response were sampled 7 days after inoculation. Collected samples were immediately frozen in liquid nitrogen before being stored at −80 °C prior to analysis. Wheat spikes were ground using a TissueLyser (Qiagen Retsch GmbH, Hannover, Germany) for 1 min at 30 Hz. A fine powder obtained was weighed into microtubes for further analysis.

### 4.4. Determination of Lipid Peroxidation Level

The level of lipid peroxidation in wheat spikes was determined by measuring the concentration of reactive substances of thiobarbituric acid (TBARS), mainly malondialdehyde (MDA), according to the method of Verma and Dubey [41]. About 150 mg of wheat spikes tissue was homogenized on ice with 1 mL of 0.1% (w/v) solution of trichloroacetic acid (TCA) and centrifuged at 6000× *g* for 5 min. To an aliquot (0.5 mL) of the supernatant, 1 mL of 0.5% thiobarbituric acid in 20% TCA was added, and the mixture was incubated in a water bath at 95 °C for 30 min. The produced red pigment was measured spectrophotometrically at 532 nm and 600 nm. The absorbance at 600 nm is deducted from the absorbance at 532 nm due to the correction for a non-specific reaction. Results were expressed in nmol of TBARS per gram of fresh weight (nmol TBARS g^–1^ FW).

### 4.5. Determination of the Soluble Phenolic Content

Powdered wheat tissue was homogenized on ice with 1 mL of 80% ethanol (1:10, w/v) and phenolic compounds were extracted for 24 h at −20 °C. After extraction, samples were centrifuged at 21,000× *g* at 4 °C for 15 min and supernatant was used for further measurements. Soluble phenolic content was determined by the Folin–Ciocalteu method [42]. The reaction mixture contained 20 μL of sample, 1.58 mL of H_2_O, 100 μL of Folin–Ciocalteu reagent, and 300 μL of the saturated Na_2_CO_3_ solution. The reaction mixture was incubated in a water bath at 37 °C for 60 min, after which the absorbance was measured at 765 nm. Soluble phenolic content was calculated from a standard curve using gallic acid as a standard and expressed as μg gallic acid equivalents (GAE) per g^−1^ fresh weight (μg GAE g^–1^ FW).

### 4.6. Enzyme Activities

A fine powder obtained after grinding was homogenized on ice in cold 100 mM potassium phosphate buffer (1:5, w/v) containing 1 mM ethylenediaminetetraacetic acid (EDTA), and 0.2% (w/v) polyvinylpyrrolidone (PVP), pH 7.0. The homogenized samples were then centrifuged at 21,000× *g* at 4 °C for 15 min, and the supernatants were used for the spectrophotometric determination of the activity of the enzymes catalase (CAT), ascorbate peroxidase (APX), and glutathione reductase (GR). Additionally, protein concentration in the enzyme extracts was determined using bovine serum albumin as a protein standard [43].

Briefly, catalase (CAT, EC 1.11.1.6) activity was measured according to Aebi [44]. The reaction mixture (1.5 mL) consisted of enzyme extract (50 μL) and 0.036% in 50 mM potassium phosphate buffer (pH 7.0). The decrease in absorbance due to the oxidation of H_2_O_2_ was monitored at 240 nm over 2 min. The CAT activity was calculated using a molar extinction coefficient (ε = 0.04 mM/cm) and expressed in U mg^−1^ protein.

Ascorbate peroxidase (APX, EC 1.11.1.11) activity was measured by an adjusted method from Nakano and Asada [45]. The reaction mixture (1 mL) consisted of enzyme extract (50 μL), 0.5 mM ascorbic acid, 0.12 mM H_2_O_2_ and 0.1 mM EDTA in 50 mM potassium phosphate buffer (pH 7.0). The decrease in absorbance due to the oxidation of ascorbate was monitored at 290 nm every 15 s for 3 min. The APX activity was calculated using a molar extinction coefficient (ε = 2.8 mM/cm) and expressed in U mg^−1^ protein.

Glutathione reductase (GR, EC 1.6.4.2) activity was measured according to Halliwell and Foyer [46]. The reaction mixture (1 mL) consisted of protein extract (50 μL), 1 mM oxidized glutathione (GSSG), 0.1 mM reduced form of nicotinamide adenine dinucleotide phosphate (NADPH) and 1 mM EDTA in 100 mM potassium phosphate buffer (pH 7.5). A decrease in absorbance due to the oxidation of NADPH was monitored at 340 nm every 15 s for 2 min. The GR activity was calculated using a molar extinction coefficient for NADPH (ε = 6.220 mM/cm) and expressed in U mg^−1^ protein.

### 4.7. Determination of Photosynthetic Pigment Concentration

A fine powder obtained after grinding (about 100 mg) was homogenized on ice with the cold absolute acetone and reextracted until plant tissue was completely colorless. The samples were centrifuged at 21,000× *g* at 4 °C for 15 min, and the supernatants were used for further measurements. The absorption of extracted photosynthetic pigments was measured at 470, 645 and, 662 nm. Concentrations of photosynthetic pigments were calculated according to Lichtenthaler [47] and expressed as mg g^−1^ fresh weight.

### 4.8. Statistical Analysis

All data analyses were performed using the SAS Enterprise Guide 7.1 (SAS Institute Inc., Cary, NC, USA) software. Assays were carried out in six replicates and their results were expressed as mean ± standard deviation (SD). Factorial analysis of variance (ANOVA) was performed, and statistically significant differences among the treatments in each variety separately were determined using the Fisher’s LSD test (*p* ≤ 0.05).

## Figures and Tables

**Table 1:** Three-way ANOVA for visual scoring and five different biochemical parameters under different nitrogen and *Fusarium* treatments in spikes of nine winter wheat varieties.

				MS			
Source of Variation	df	VS^2^	TBARS	PHE	CAT	APX	GR
VARIETY (V)	8	0.08 ***	59.92 ***	108.02 ***	1700.63 ***	1.295 ***	719.05 ***
N LEVEL (N)	1	0.04 *	43.73 **	20.51 ns	1215.25 **	4.227 ***	180.06 ns
FUSARIUM (F)	1	23.02 ***	11.62 ns	190.55 ***	669.20 *	0.001 ns	733.05 *
V×N	8	0.14 ***	17.14 ***	38.75 *	163.39 ns	0.571 ***	176.72 ns
V×F	8	0.08 ***	6.78 ns	25.96 ns	801.97 ***	0.307 ***	299.44 ns
N×F	1	0.04 *	2.31 ns	153.85 **	1769.33 ***	0.338 *	808.47 *
V×N×F	8	0.14 ***	7.16 ns	27.53 ns	579.37 ***	0.439 ***	321.27 ns

ns—not significant, *, ** and ***—significant at the level of probability *p* ≤ 0.05, 0.01, and 0.001, respectively. Df, degrees of freedom; MS, mean sum of squares; VS, visual scoring (in percentage inoculated spikes); TBARS, thiobarbituric acid reactive substances; PHE, phenolics; CAT, catalase; APX, ascorbate peroxidase; GR, glutathione reductase. ^2^ A log transformation of the visual scores was used to normalize the data.

**Table 2:** Visual scores for Fusarium head blight (FHB) of nine inoculated winter wheat varieties under two different nitrogen levels.

	Visual Scores (in Percentage Inoculated Spikes)								
N level	Ficko	U-1	Galloper	BC Mandica	BC Opsesija	Ingenio	Isengrain	Felix	Bezostaya-1
LN	6.67 ± 1.53 b	8.00 ± 1.00 a	3.33 ± 1.53 b	9.00 ± 5.29 a	20.00 ± 10.00 a	10.33 ± 4.51 a	10.00 ± 0.00 a	8.33 ± 2.89 b	5.00 ± 0.00 a
HN	25.00 ± 5.00 a	3.33 ± 0.58 b	10.00 ± 0.00 a	7.00 ± 2.65 a	4.33 ± 0.58 a	10.33 ± 4.51 a	8.33 ± 2.89 a	32.67 ± 2.52 a	8.00 ± 2.00 a

Values are means of three replicates ± standard deviation (SD). Different letters indicate significant differences according to Fisher’s LSD test (*p* ≤ 0.05) among different nitrogen treatments in each variety separately. LN—low nitrogen; HN—high nitrogen.

**Table 3:** Three-way ANOVA for different chloroplast pigments under different nitrogen and *Fusarium* treatment in spikes of nine winter wheat varieties.

				MS		
Source of Variation	df	Chl a	Chl b	Chl a+b	Car	Chl a/Chl b
VARIETY (V)	8	0.0128 ***	0.0015 ***	0.0228 ***	0.0008 ***	0.0745 **
N LEVEL (N)	1	0.0363 ***	0.0058 ***	0.0712 ***	0.0019 ***	0.1192 *
FUSARIUM (F)	1	0.0204 ***	0.0033 ***	0.0402 ***	0.0014 ***	0.0592 ns
V × N	8	0.0011 ns	0.0002 ns	0.0022 ns	0.0001 ns	0.0217 ns
V × F	8	0.0025 *	0.0004 *	0.0050 *	0.0002 ns	0.0258 ns
N × F	1	0.0028 ns	0.0003 ns	0.0049 ns	0.0002 ns	0.0059 ns
V × N × F	8	0.0049 ***	0.0008 ***	0.0097 ***	0.0003 ***	0.0229 ns

ns—not significant, *, ** and ***—significant at the level of probability *p* ≤ 0.05, 0.01, and 0.001, respectively. Df, degrees of freedom; MS, mean sum of squares; Chl a, chlorophyll a; Chl b, chlorophyll b; Chl a+b, total chlorophyll; Car, carotenoids; Chl a/Chl b, chlorophyll a/b ratio.

**Table 4:** Winter wheat varieties and their origin.

Variety	Country	Breeding Institution	Year of Release
BC Mandica	Croatia	Bc Institut d.d. Zagreb	2015
BC Opsesija	Croatia	Bc Institut d.d. Zagreb	2016
Bezostaya-1	Russia	Krasnodar Lukyanenko Research Institute	1959
Felix	Croatia	Agricultural Institute Osijek	2007
Ficko	Croatia	Agricultural Institute Osijek	2007
Galloper	Croatia	Agricultural Institute Osijek	2014
Ingenio	France	CC Benoist SA	2010
Isengrain	France	Florimond Desprez Veuve et Fils (FR)	1997
U-1	Croatia	Agricultural Institute Osijek	1936

**Table 5:** Soil nitrogen (N) content (kg ha^−1^) in Osijek in 2017/18 year.

Location	Soil Type	Season	Residual Soil N (kg N ha^−1^)	Basic N Fertilization (kg N ha^−1^)	N Top-Dressing (kg N ha^−1^)		Total N (kg N ha^−1^)	
					LN	HN	LN	HN
Osijek	Eutric cambisol	2017/18	20	74	0	50 + 50	94	194

## Data Availability

The dataset used and/or analyzed during the current study are available from the corresponding authors on reasonable request.
